# Paper Electrodes Coated with Partially-Exfoliated Graphite and Polypyrrole for High-Performance Flexible Supercapacitors

**DOI:** 10.3390/polym10020135

**Published:** 2018-01-31

**Authors:** Leping Huang, Weida Rao, Lingling Fan, Jie Xu, Zikui Bai, Weilin Xu, Haifeng Bao

**Affiliations:** 1 State Key Laboratory for Hubei New Textile Materials and Advanced Processing Technology, School of Materials Science & Engineering, Wuhan Textile University, Wuhan 430200, China; hlp71@126.com (L.H.); weidayumiko@163.com (W.R.); zkbwn@163.com (Z.B.); weilin-xu@hotmail.com (W.X.); 2 School of Textile Science & Engineering, Wuhan Textile University, Wuhan 430200, China; hglingling@126.com

**Keywords:** paper, polypyrrole, partially-exfoliated graphite, supercapacitor

## Abstract

Flexible paper electrodes for supercapacitors were prepared with partially-exfoliated graphite and polypyrrole as the active materials. Graphite was coated on paper with pencil drawing and then electrochemically exfoliated using the cyclic voltammetry (CV) technique to obtain the exfoliated graphite (EG)-coated paper (EG-paper). Polypyrrole (PPy) doped with β-naphthalene sulfonate anions was deposited on EG-paper through in-situ polymerization, leading to the formation of PPy-EG-paper. The as-prepared PPy-EG-paper showed a high electrical conductivity of 10.0 S·cm^−1^ and could be directly used as supercapacitor electrodes. The PPy-EG-paper electrodes gave a remarkably larger specific capacitance of 2148 F∙g^−1^ at a current density of 0.8 mA∙cm^−2^, compared to PPy-graphite-paper (848 F∙g^−1^). The capacitance value of PPy-EG-paper could be preserved by 80.4% after 1000 charge/discharge cycles. In addition, the PPy-EG-paper electrodes demonstrated a good rate capability and a high energy density of 110.3 Wh∙kg^−1^ at a power density of 121.9 W∙kg^−1^. This work will pave the way for the discovery of efficient paper-based electrode materials.

## 1. Introduction

Flexible electronics have recently attracted considerable attention because of their potential applications in rollable displays, e-paper, smart textiles, distributed sensors, radio frequency identification, embedded health monitoring devices, and so on [1,2,3,4,5,6]. As an important component to power the functional devices, there is a crucial challenge to develop lightweight, flexible, and high-performance energy storage devices [6,7,8]. Flexible supercapacitors have been one of the most promising energy storage devices due to their high power density, long cycle life, and fast charge-discharge rate [9]. Based on the energy storage mechanisms, supercapacitors can be divided into two main types, namely electrical double-layer capacitors (EDLCs) and pseudocapacitors. EDLCs are electrostatic storage of the electrical energy done by separating the charge in a double layer at the interface between electrode and electrolyte, while pseudocapacitors provide electrochemical storage of the electrical energy, achieved by faradic redox reactions.

Polypyrrole (PPy), a representative conducting polymer, was widely used as a pseudocapacitive electrode material due to its low cost, high theoretical specific capacitance, superior electrical conductivity, fast redox activity, appreciable mechanical property, and facile preparation [10,11,12,13,14,15,16,17,18,19,20,21]. However, most PPy-based electrodes retain less than 50% of the initial capacitance after 1000 charge/discharge cycles [22], which is not acceptable for supercapacitors. The charge/discharge cycling instability of PPy is mainly due to structural deformation and counter-ion drain effect [23]. The backbone of the PPy chains experiences repeated swelling and shrinking during cycling as a result of doping and dedoping of counter-ions, leading to structural pulverization and, thus, fast capacitance decay. Moreover, some of the ion channels collapse when anions diffuse back to the electrolyte, making anions more difficult to re-dope the PPy backbone. Thus, the conductivity and capacitance of PPy would reduce slowly. Extensive efforts have been devoted to improve the cycling stability of PPy, including integrating PPy into flexible substrates, such as graphene [24] and carbon nanotubes (CNTs) [25], and doping PPy with special anionic dopants [23,26,27].

Paper, as one of the most available flexible products, is not only used for information recording and packaging, but has also been considered as a promising flexible substrate for energy storage devices due to its wide availability, low cost, light weight, environmental friendliness, recyclability, and bendability [6,28]. Zheng et al. used a pure graphite rod to draw on Xerox cellulose paper to obtain a solvent-free drawing electrode [29]. An aqueous supercapacitor based on the pencil-drawing paper electrodes can deliver a specific capacitance of 12 F∙g^−1^ at a current density of 10 mA∙g^−1^ and retain 90% its initial capacitance after 15,000 cycles. The device displays excellent cycling stability; however, its specific capacitance is still relatively low. Later, Tao et al. used an electrochemical deposition method to grow PPy on the pencil-drawing graphite paper [30]. A solid-state supercapacitor fabricated with the PPy/graphite coated paper achieved a high volumetric specific capacitance of 52.9 F∙cm^−3^ at a scan rate of 1 mV∙s^−1^.

Functionalized exfoliated graphite (EG) has been demonstrated to offer a large surface area for supporting pseudo-capacitive materials [31]. The strong attachment of functionalized graphene on the graphite base could minimize the interfacial contact resistance and, thus, improve the rate capability of pseudo-capacitive electrodes. In this work, we prepared flexible paper electrodes coated with EG and PPy. Graphite was coated on paper with pencil drawing and then electrochemically exfoliated to obtain the EG coated paper (EG-paper). Polypyrrole (PPy) doped with β-naphthalene sulfonate anions was deposited on EG-paper. The as-prepared PPy-EG-paper electrode delivered an enhanced specific capacitance, superior cycling stability, and good rate capability, thereby making it a viable electrode material for flexible supercapacitors.

## 2. Materials and Methods

### 2.1. Materials

Pyrrole, cetyltrimethylammonium bromide (CTAB), sodium dodecyl benzene sulfonate (SDBS), β-naphthalene sulfonic acid, and other chemicals were purchased from Sinopharm Chemical Reagent Co., Ltd. (Shanghai, China) and used as received. Deionized water (18.2 MΩ∙cm^−1^) was obtained from a Milli-Q water-purification system.

### 2.2. Preparation of PPy-EG-paper

A piece of Sketch paper (Maxleaf, Jianli, China) with an area of about 2.0 cm^2^ was drawn with a 4B pencil (Zhonghua, Shanghai, China) 50 times to form a continuous graphite layer onto the paper. Pencil drawing provides a rapid and extremely cost-effective approach for the production of electrochemical platforms. However, the number of layers affects the electrochemical signal dramatically [32,33]. The graphite layer could be screen-printed on the paper by using graphite paste for repeatability and reproducibility. The graphite-coated paper (G-paper) was used as the working electrode in a three-electrode electrochemical system with saturated calomel electrode (SCE) as the reference electrode and platinum (Pt) foil as the counter electrode. The G-paper electrode was scanned on an Autolab PGSTAT302N potentiostat/galvanostat system (Metrohm AG, Herisau, Switzerland) for six cycles between 0.5 and 1.8 V versus SCE at a scan rate of 20 mV∙s^−1^ in 0.5 M K_2_CO_3_ aqueous solution. The G-paper electrode was then further scanned for another ten cycles between −1.0 and 1.9 V versus SCE at 20 mV∙s^−1^ in phosphate-buffered saline containing 1 M KNO_3_ (pH = 6.7). In each cycle, the G-paper electrode was kept at 1.9 V versus SCE for 5 s. The paper was cleaned with deionized water and ethanol and vacuum-dried at ambient temperature. Thus, the exfoliated graphite-coated paper (EG-paper) was obtained.

PPy was deposited on the EG-paper via in-situ polymerization as previously reported [13,14,15]. Briefly, the EG-paper was dipped into 100 mL aqueous solution containing pyrrole (1.0 M), CTAB (0.002 M), and SDBS (0.002 M) and stirred for 30 min. Then, 100 mL aqueous solution of FeCl_3_ (0.5 M) was slowly added to the mixture under stirring. The mixture was stirred for another 2 h at 5 °C. The resulting paper was then immersed in 0.5 M aqueous solution of β-naphthalene sulfonic acid for 30 min. The paper was brought out and washed with deionized water and ethanol, in sequence, three times. Finally, the paper was dried at 60 °C overnight. The thus-obtained PPy-coated EG-paper was denoted as PPy-EG-paper.

For comparison, the PPy-coated G-paper (denoted as PPy-G-paper) was also prepared according to the same procedure using the G-paper substrate as a control sample.

### 2.3. Characterization

Scanning electron microscopy (SEM, JEOL JSM-6510LV, Tokyo, Japan, operated at 10 kV) was used to investigate the morphology. Raman spectra were obtained with a Renishaw microRaman spectroscopy system (633 nm, Renishaw, Derbyshire, UK). The Brunauer–Emmett–Teller (BET) surface area was measured on a TriStar II 3020 analyzer (Micromeritics Instrument Corp., Norcross, GA, USA). Attenuated total reflection Flourier transformed infrared (ATR-FTIR) spectra were recorded on a Tensor 27 (Bruker, Ettlingen, Germany) in the range of 4000–600 cm^−1^. Surface conductivity was determined on a digital four-point probe resistivity measurement system (RTS-9, Probes Tech. Co., Shenzhen, China).

Electrochemical measurements were carried out via a two-electrode symmetric supercapacitor system. The sample and mass of positive and negative electrodes are exactly the same. The coated paper was directly used as the electrodes, and the electrolyte was a 2.0 M NaCl aqueous solution. Cyclic voltammetry (CV) curves were collected within the voltage range of −0.9–+0.9 V at various scan rates (5 to 100 mV∙s^−1^) on an Autolab PGSTAT302N. Galvanostatic charge-discharge (GCD) cycling measurements were conducted over the potential range from 0 to 0.8 V on a multichannel LAND CT2001A battery testing device (Wuhan Land Electronics, Wuhan, China). The mass of the active material was determined by weight measurements before and after the coating of the active material.

## 3. Results and Discussion

**Figure 1:**
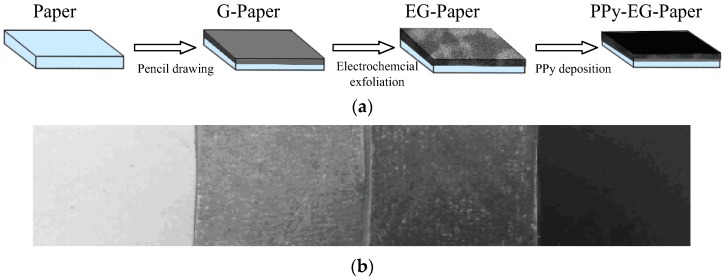
(**a**) Schematic of fabrication process of the PPy-EG-paper; and (**b**) photographs of the blank paper, G-paper, EG-paper, and PPy-EG-paper.

**Figure 2:**
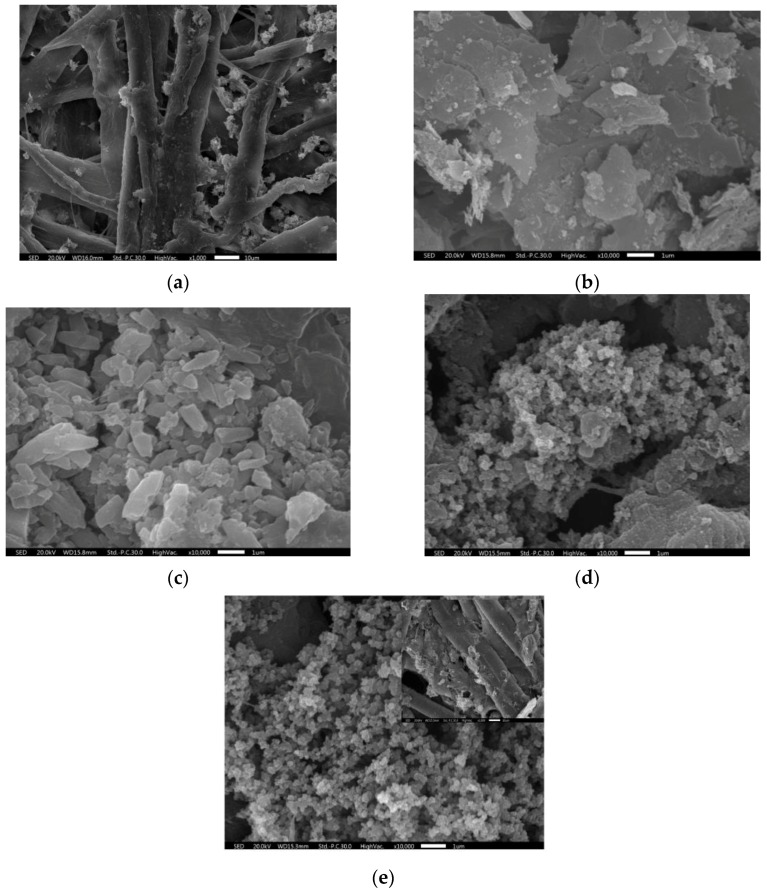
SEM images of (**a**) blank paper; (**b**) G-paper; (**c**) EG-paper; (**d**) PPy-G-paper; and (**e**) PPy-EG-paper.

The fabrication process of the PPy-EG-paper composite is illustrated in Figure 1a. The white paper blackened after the pencil drawing and turned black completely after the PPy deposition (Figure 1b). The paper mainly consists of cellulose fibers (Figure 2a). It can be seen from Figure 2b that the lamellar graphite was produced and coated on the paper substrate with pencil drawing. The graphite was partially electrochemically exfoliated using the CV method and the graphene multilayers were formed in small sizes (dimensions of one to a few microns, Figure 2c). The BET surface area of EG-paper was about 3.91 m^2^/g, which is higher than that of G-paper (3.01 m^2^/g). The enlarged surface area could support the deposition of PPy. The structure of EG could maintain the electronic connections between graphene sheets through the graphite matrix, which will facilitate electron transfer and benefit electrochemical reactions [34]. After in situ polymerization, PPy was successfully wrapped on the G-paper and EG-paper (Figure 2d,e). The PPy coatings were agglomerated into lumps with different sizes. The PPy particles in the PPy-EG-paper composites were smaller in size and more regular in shape as compared with those in the PPy-G-paper. The PPy particles were stacked to form a three-dimensional interconnected network, which could be beneficial to enhance the electrolyte adsorption and expand the ion transport channels.

**Figure 3:**
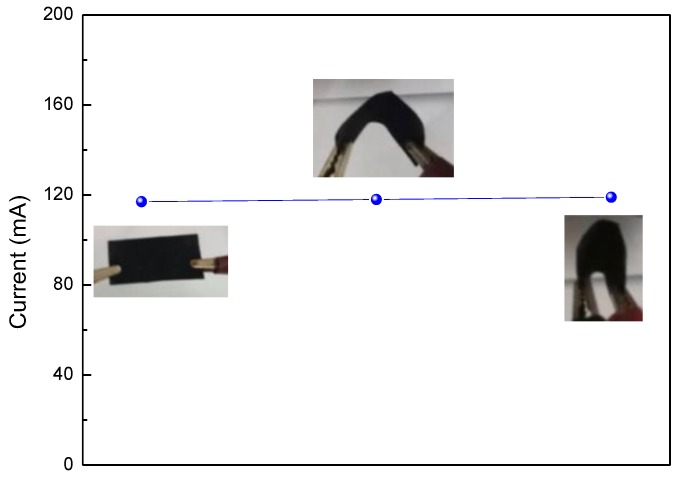
The current PPy-EG-paper bent with different curvatures under a constant voltage of 0.8 V.

The mass loading of PPy of the total weight of the composite paper was about 9.5% and 6.5% for PPy-G-paper and PPy-EG-paper, respectively. The electrical conductivity was about 3.3 and 10.0 S∙cm^−1^ for PPy-G-paper and PPy-EG-paper, respectively. The conductivity of PPy-EG-paper is higher than those of PPy-coated Cladophora paper (about 1 S∙cm^−1^) [35], PPy/MWCNTs/bacterial cellulose (BC) composite paper (7.78 S·cm^−1^) [36], PPy/BC paper (3.9 S·cm^−1^) [12], and pure PPy (1.14 S·cm^−1^) [37]. In order to demonstrate the suitability of PPy-EG-paper for flexible electrode materials, the conductance stability of PPy-EG-paper at different curvatures (Figure 3) was measured by monitoring the current at a fixed voltage of 0.8 V. It can be observed that the current was nearly unchanged at different bending states, revealing that the conductance of PPy-EG-paper is hardly affected by bending stress and, thus, confirming its promising potential in flexible electronics.

**Figure 4:**
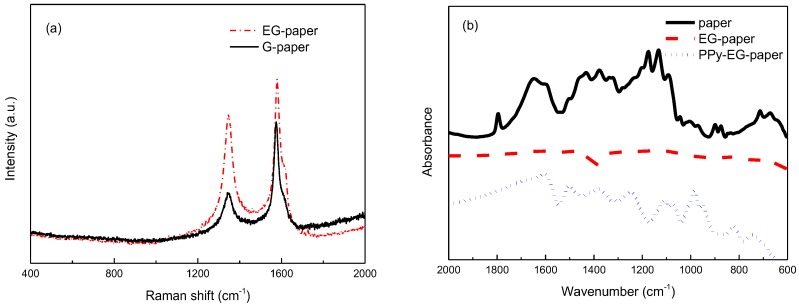
(**a**) Raman spectra of G-paper and EG-paper; and (**b**) FTIR spectra of the blank paper, EG-paper, PPy-EG-paper.

Raman spectra of G-paper and EG-paper are shown in Figure 4a. For both G-paper and EG-paper, the spectra exhibited the D (1346 cm^−1^) and G (1579 cm^−1^) bands. The D band represents the breathing vibration modes of sp^2^ carbon atoms. The G band represents the in-plane vibration modes of sp^2^ carbon atoms. The relative intensity of the D to G bands for EG-paper was larger than that for G-paper, confirming the exfoliation of graphite. The blank paper, EG-paper, and PPy-EG-paper were characterized by ATR-FTIR and the results are shown in Figure 4b. The spectrum of the blank paper was featured by the typical overlapping bands of the functional groups (C–O, C–C, and C–O–C) of cellulose at 1157, 1103, and 1022 cm^−1^. These bands disappeared for EG-paper due to the coating of EG. In the case of PPy-EG-paper, the main peaks corresponding to PPy [38,39] could be observed. The band between 1550 and 1460 cm^−1^ was due to the C=C stretching vibration in pyrrole ring. The C–N stretching vibration was located at 1378 cm^−1^. The band at 1250 cm^−1^ was assigned to the C–H and C–N in-plane deformation vibrations. The C–H bending vibration was located at 1111 cm^−1^. The peak at 1077 cm^−1^ was attributed to the in-plane deformation vibration of NH_2_^+^ groups in the protonated PPy chains. The N–H in-plane deformation vibration band was found at 985 cm^−1^.

**Figure 5:**
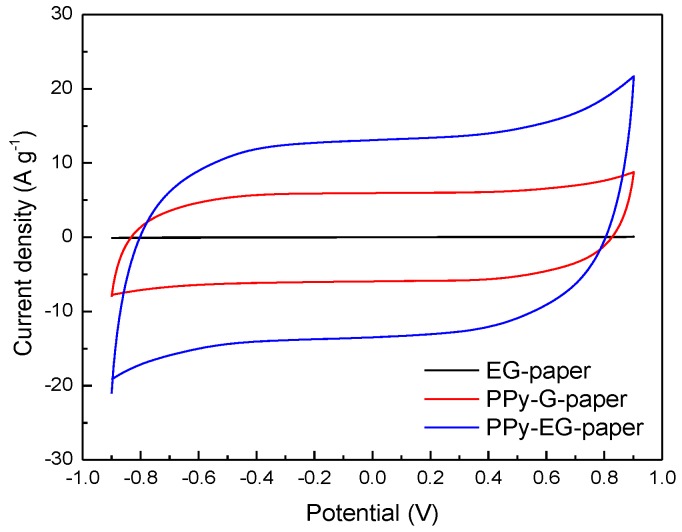
CV curves of EG-paper, PPy-G-paper, and PPy-EG-paper at a scan rate of 5 mV∙s^−1^.

To investigate the electrochemical properties of the as-prepared PPy-EG-paper, symmetrical supercapacitor devices were fabricated and subjected to CV measurements in a voltage window of −0.9–+0.9 V using a NaCl aqueous electrolyte. The CV curves of EG-paper, PPy-G-paper, and PPy-EG-paper with a scan rate of 5 mV∙s^−1^ are shown in Figure 5. Notably, the current densities of PPy-G-paper and PPy-EG-paper were significantly larger than that of EG-paper, indicating a significant contribution of pseudocapacitance by PPy. Both the CV curves of PPy-G-paper and PPy-EG-paper exhibited a quasi-rectangular shape without obvious redox peaks, implying their good capacitive behavior. Furthermore, PPy-EG-paper possessed the larger enclosed area on the CV curve than PPy-G-paper, demonstrating higher specific capacitance.

**Figure 6:**
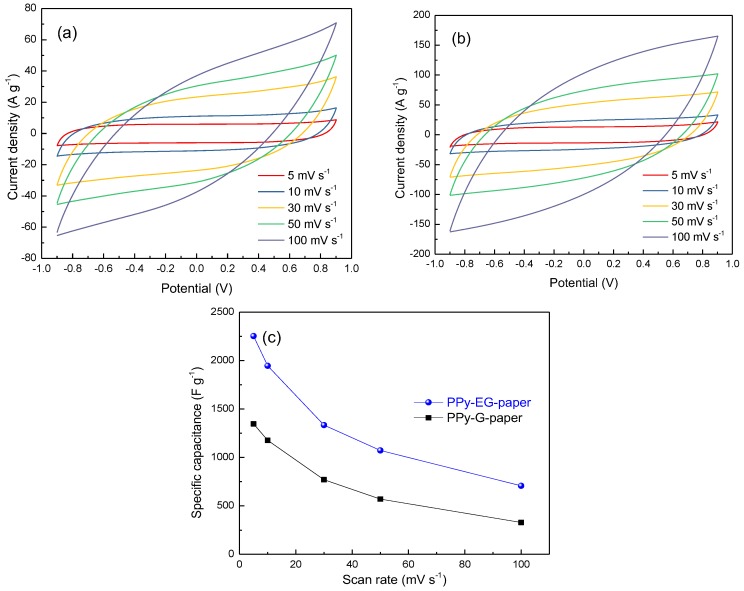
CV curves of (**a**) PPy-G-paper and (**b**) PPy-EG-paper at different scan rates; and (**c**) specific capacitance of PPy-G-paper and PPy-EG-paper at different scan rates.

The CV curves of PPy-G-paper and PPy-EG-paper with different scan rates ranging from 5 to 100 mV∙s^−1^ are shown in Figure 6a,b. The shape of the CV curves gradually transformed from a distorted rectangle to an oval upon increasing the scan rate. This may be attributed to the inhibition of charge collection by the internal resistance of the electrode and the diffusion limitations of Na^+^ in the inner active surfaces of the electrode [40]. The mass-specific capacitance can be calculated from the CV curves using the following equation:

$$C_{sp}=\frac{\int IdV}{f\times \Delta V\times m}$$

where *I* is the current, *V* is the voltage, *f* is the scan rate of the CV curves, Δ*V* is the whole voltage difference, and *m* is the mass of the electroactive material. The obtained mass-specific capacitances as a function of the scan rate for PPy-G-paper and PPy-EG-paper are shown in Figure 6c. The specific capacitance of PPy-G-paper and PPy-EG-paper was calculated from the CV curves to be 1347 and 2253 F∙g^−1^, respectively, at a low scan rate of 5 mV∙s^−1^. It can be found that the capacitance of PPy-G-paper and PPy-EG-paper was decreased as the scan rates increased, which is explained by the ion diffusion effect in the electrode materials and at the electrode/electrolyte interface [41]. Interestingly, even at a high sweep rate of 50 mV∙s^−1^, the capacitance of PPy-EG-paper remained relatively high (i.e., 1073 F∙g^−1^). It is obvious that the capacitance of PPy-EG-paper was higher than that of PPy-G-paper at all scan rates. These results indicate that PPy-EG-paper exhibited a good rate capability upon increasing the scan rate. Furthermore, the specific capacitance values of PPy-EG-paper are significantly higher than those of other recently-reported paper electrodes, such as MnO_2_/Ni/graphite/paper (680 F∙g^−1^ at 5 mV∙s^−1^) [42], Mn_3_O_4_/Ni/graphite/paper (432 F∙g^−1^ at 5 mV∙s^−1^) [43], PANI/nylon/cellulose acetate (400 F∙g^−1^ at 5 mV∙s^−1^) [44], CNTs/MnO_2_/CNTs/paper (327 F∙g^−1^ at 10 mV∙s ^−1^) [45], and MnO_2_/MWCNTs/paper (1035 F∙g^−1^ at 2 mV∙s^−1^) [46].

**Figure 7:**
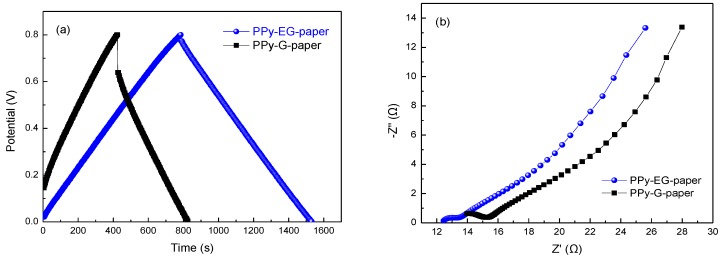
(**a**) GCD curves of PPy-G-paper and PPy-EG-paper at a current density of 0.8 mA∙cm^−2^; and (**b**) Nyquist plots of PPy-G-paper and PPy-EG-paper.

**Figure 8:**
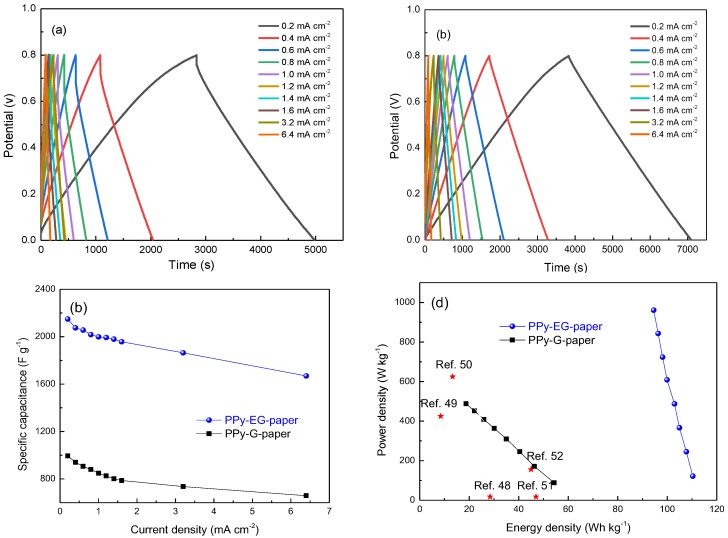
GCD curves of (**a**) PPy-G-paper and (**b**) PPy-EG-paper at different current densities; (**c**) specific capacitance of PPy-G-paper and PPy-EG-paper at different current densities; and (**d**) Ragone plots of PPy-G-paper and PPy-EG-paper.

The GCD curves of PPy-G-paper and PPy-EG-paper at the same current density of 0.8 mA∙cm^−2^ are illustrated in Figure 7a. The curve of PPy-EG-paper had symmetrical triangle and small internal resistance drop (*IR_drop_*), demonstrating the superior reversibility of charging/discharging. The equivalent series resistance due to electrolyte resistance, electrode resistance, and contact resistance might be responsible for this *IR_drop_*. The *IR_drop_* of PPy-EG-paper was lower than that of PPy-G-paper, which is in agreement with the conductivity results. Electrochemical impedance spectroscopy (EIS) was used to further understand the interfacial resistance for PPy-EG-paper and PPy-G-paper. The Nyquist plots are displayed in Figure 7b. The equivalent resistance for PPy-EG-paper was smaller than that for PPy-G-paper, indicating the superior performance of PPy-EG-paper. The specific capacitance (*C_m_*) could be determined based on the GCD profile using the following formula:

$$C_{m}=\frac{I\times t}{(\Delta V-IR_{drop})\times m}$$

where *I* is the discharge current, *t* is the time elapsed for the discharge from 0 to 0.8 V, Δ*V* is the voltage difference within the discharge time, *IR_drop_* is the IR voltage drop, and *m* is the mass of the active materials on the electrode. The specific capacitance at 0.8 mA·cm^−2^ was calculated to be about 848 and 2148 F∙g^−1^ for PPy-G-paper and PPy-EG-paper, respectively. The specific capacitance of PPy-EG-paper was significantly higher than that of PPy-G-paper, which is in agreement with the trend revealed by the CV curves. This value is even higher than the proposed results of PPy-coated paper electrodes (370 F∙g^−1^ at 1 mA∙cm^−2^) [47], PANI/Au/paper electrodes (560 F∙g^−1^ at 1.0 A∙g^−1^) [48], graphene nanosheet/cellulose composite paper electrodes (252 F∙g^−1^ at 1 A∙g^−1^) [49], PANI/cellulose paper electrodes (160 F∙g^−1^ at 0.1 A∙g^−1^) [28], N/S-codoped graphene paper electrodes (281 F∙g^−1^ at 1 A∙g^−1^) [50], Mn_3_O_4_/Ni/graphite/paper electrodes (372.5 F∙g^−1^ at 1.0 ∙m^−2^) [43], and PANI/nylon/cellulose acetate (402 F∙g^−1^ at 0.3 A∙g^−1^) [44]. The GCD curves and *C_m_* values for PPy-G-paper and PPy-EG-paper at different current densities are shown in Figure 8a–c. No obvious *IR_drop_* appeared with increasing the current density for PPy-EG-paper, indicating its lower resistance. It can be found that the *C_m_* values decreased slightly with the increase of the current densities. When the current density rose up to 6.4 mA∙cm^−2^, the specific capacitance of PPy-G-paper and PPy-EG-paper remained at 659 and 1669 F∙g^−1^, respectively, corresponding to 66.3% and 77.8% of the initial values. Specific energy density (*E*) and power density (*P*) are calculated using the following equations:

*E* = (1/2)*CV*^2^

*P* = *E*/*t*

where *C* is the specific capacitance, *V* the operating voltage and *t* the discharge time. The Ragone plots of specific power density against specific energy density for PPy-G-paper and PPy-EG-paper are shown in Figure 8d. In both cases, the obtained curves indicate that the specific power density decreased upon increasing the specific energy density. PPy-G-paper showed relatively smaller specific energy and power densities due to the lower *C_m_* values and larger IR drops during the GCD process. In the case of PPy-EG-paper, the energy density reached to about 110.3 Wh∙kg^−1^ at a power density of 121.9 W∙kg^−1^, which is remarkably higher than the reported values for paper electrodes, such as PPy-cladophora cellulose paper (1.75 Wh∙kg^−1^) [35], nitrogen/sulfur-codoped graphene paper (28.44 Wh∙kg^−1^) [50], V_2_O_5_-graphene paper (8.5 and 13.3 Wh∙kg^−1^) [51,52], T-Nb_2_O_5_-graphene paper (47 Wh∙kg^−1^) [53], V_2_O_5_-carbon fiber paper (45 Wh∙kg^−1^) [54], and CNT-paper (48.86 Wh∙kg^−1^) [55].

**Figure 9:**
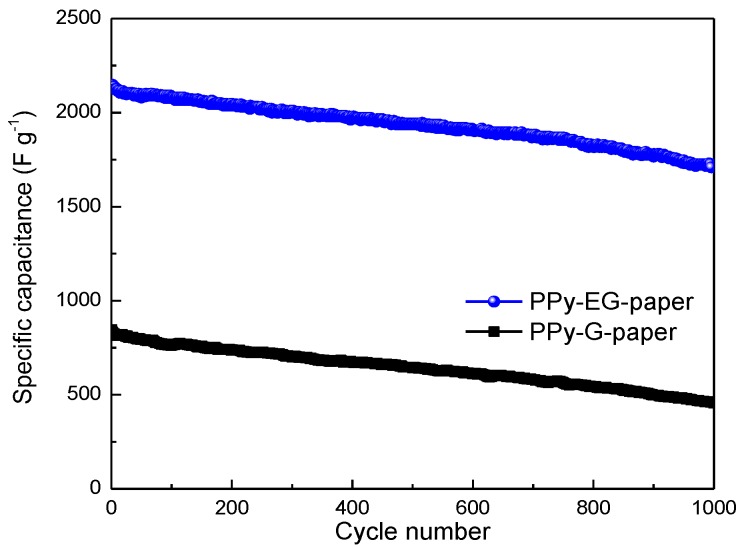
Cycling performance of PPy-G-paper and PPy-EG-paper at a current density of 0.8 mA∙cm^−2^.

Cycling life was used to comprehensively evaluate the electrochemical performance of the electrode materials. Figure 9 displays the cycling stability of PPy-G-paper and PPy-EG-paper at a current density of 0.8 mA∙cm^−2^. Notably, the downward trend of the specific capacitance for PPy-EG-paper was flat, while the specific capacitance of PPy-G-paper was sharply decreased. After 1000 cycles, the specific capacitance of PPy-EG-paper still possessed 1726 F∙g^−1^, meaning that 80.4% of its initial value was retained; however, PPy-G-paper would lessen to 54.0%. PPy-EG-paper was endowed with a long cycling life and further stable electrochemical performance. Thus, the above results indicate that the PPy-EG-paper electrodes prepared herein exhibited high specific capacitance, low ESRs, good capacitance retention, and good cycling stabilities, revealing their great potential for flexible energy storage.

## 4. Conclusions

In summary, we prepared the paper electrodes for supercapacitors with partially-exfoliated graphite and polypyrrole as the active materials. Graphite was coated on paper with pencil drawing and electrochemically exfoliated. PPy were deposited on the EG-paper through in situ polymerization. The as-prepared PPy-EG-paper exhibited remarkable electrochemical performance, in terms of a high specific capacitance of 2148 F∙g^−1^ at a current density of 0.8 mA∙cm^−2^, a high energy density of 110.3 Wh·kg^−1^ at a power density of 121.9 W·kg^−1^, favorable rate capability, and improved cycling life with retention of the initial specific capacitance at 80.4% after 1000 charge/discharge cycles. These results demonstrate the potential of PPy-EG-paper as a promising electrode for flexible supercapacitors.
